# Assessing vulnerabilities and resilience strategies for communities facing climate change in Androy, Southern Madagascar

**DOI:** 10.3389/fpubh.2026.1747679

**Published:** 2026-04-09

**Authors:** Aline Mutabazi, Charles Mahafake, Ami Fall, Angali Ramnarayan, Maya Moukarzel, Mohd Faizaan Khan, Chichi Otti, Yifeng Liang, Cara Rubin, Nirina Holongoe, Merci Mahatradraza, Liasinorie Kazy, Emmanuel Todisoa Miandrireny Tsiorendraha, Sylvain Mahazotahy, Chris Dickey

**Affiliations:** 1 Department of Global Public Health, New York University, New York, NY, United States; 2 Central Androy Regional University, Ambovombe-Androy, Madagascar

**Keywords:** climate change, climate health, climate resilience, food insecurity, Madagascar

## Abstract

The Androy region of southern Madagascar faces compounding challenges of climate change, environmental degradation, and socioeconomic instability. These challenges have forced local communities confronting prolonged droughts, food insecurity, and ecosystem collapse to develop a range of adaptive strategies and resilience mechanisms. Using a cross-sectional mixed-methods design, researchers from the Centre Universitaire Régional Androy (CURA) and New York University's Applied Global Public Health Initiative (AGPHI) conducted 16 focus groups and community surveys across five rural districts to better understand and evaluate these adaptive strategies. The data was analyzed through Dice–Sørensen similarity coefficients. Four distinct adaptation profiles: community-driven reforestation, educational initiatives, reliance on humanitarian aid, and the adoption of drought-resistant crops and short-cycle agriculture. The study highlights the need for integrated, community-centered adaptation strategies that strengthen health systems, expand climate literacy, and promote sustainable agriculture practices. It further provides a scalable framework for addressing climate-induced vulnerabilities in similar semi-arid, resource-constrained contexts.

## 1 Introduction

Climate change poses an escalating threat to public health worldwide. Since 1975, global surface temperatures have risen by an average of 1.1 degrees Fahrenheit, reaching record highs in 2024, triggering more frequent and extreme weather events, and significantly impacting human lives (1, 2). Climate change has been linked to a notable increase in mortality among people over the age of 65, as well as detrimental effects on sleep quality, physical health, and mental health (1).

Climate-related disasters, such as droughts and heatwaves, have heightened the risk of moderate to severe food insecurity by reducing crop yields or depleting marine resources (1, 2).

Madagascar is one of the countries most impacted by climate change. The island, located off the east coast of Africa, is rich in biodiversity and a range of environments, from tropical rainforests in the east to spiny deserts in the south (3). Although Madagascar is known for its rich natural diversity, it remains one of the poorest countries in the world, with a population primarily dependent on environmental resources for survival (4). The country's major economic sectors, including fishing, agriculture, and livestock production, are directly impacted by climate change, heightening poverty, and food insecurity. The southern region of Madagascar bears the brunt of its detrimental effects (2, 5). This region is marked by political neglect, which has exacerbated the lack of infrastructure and contributed to a significant nutritional crisis (6). Multiple climate factors, including cyclones, floods, severe droughts, and sandstorms, have significantly limited access to critical food resources. As a result, famine and child malnutrition continued to rise, particularly in areas facing significant food insecurity. Of the rural populations, 12% suffer from acute food insecurity, with the most affected districts being Ambovombe Androy and Amboasary Atsimo (2, 7). Previous research done on the impacts of climate change in the Androy region of Madagascar is limited in comparison to other regions on the island. To understand the gravity of climate change's impact on the country, the most rural, underserved regions must be studied in collaboration with local communities.

The Androy region is part of the Ambovombe district, characterized by semi-arid environments and thorny landscapes (8). This region is known as “Androy, where the water hides,” and the population of Androy is referred to as the Ntandroy. Climate change has caused the Ntandroy to experience a hot, dry climate with limited agricultural produce, making it essential for communities to adopt adaptation strategies to ensure adequate food consumption (9). The adverse conditions brought forth by droughts pose significant risks to agriculture, a critical local economic activity. These conditions lead to decreased agricultural yields, contributing to high famine and malnutrition. These environmental changes and prolonged droughts have resulted in catastrophic starvation events known as “Kere,” which deeply impact the most impoverished communities (10).

The natural hazards have significantly altered the lives of the Ntandroy population. Droughts have forced most individuals to adapt their diets during long stretches between harvests, leading them to rely on tubers, wild fruits, and small mammals found in the thorny bush. Despite these food adaptations and the humanitarian aid provided in Southern Madagascar, (11) ongoing environmental degradations have made it increasingly difficult for the Ntandroy to survive during lean periods, resulting in heightened suffering from famine (12). Over the years, the Ntandroy population has been driven to adapt to climate change both culturally and socially as a means of survival. However, the current situation has increased insecurity and undermined their survival strategies. This has led to a rise in permanent migration, undermining the capacity of communities to support one another (13).

For effective climate planning, it is essential to understand the issues, culture, behaviors, and vulnerabilities to strengthen the resilience strategies the population has developed (1). A mixed-methods study was conducted in the Androy region to identify the challenges faced by communities due to climate change and the community-level adaptations that promote resilience. This study involved interviewing focus groups, which allowed exploration of community perspectives on the gaps in current adaptation strategies and suggestions for improving responses to climate impacts. The study aims to answer three questions: (1) What main issues are encountered by the communities in the Androy region due to climate change? (2) What resilience strategies have been adopted in this region to mitigate the impact of climate change? and (3) What gaps in climate adaptation can be addressed to enhance resilience for these communities?

## 2 Methods

### 2.1 Data collection

**Figure 1:**
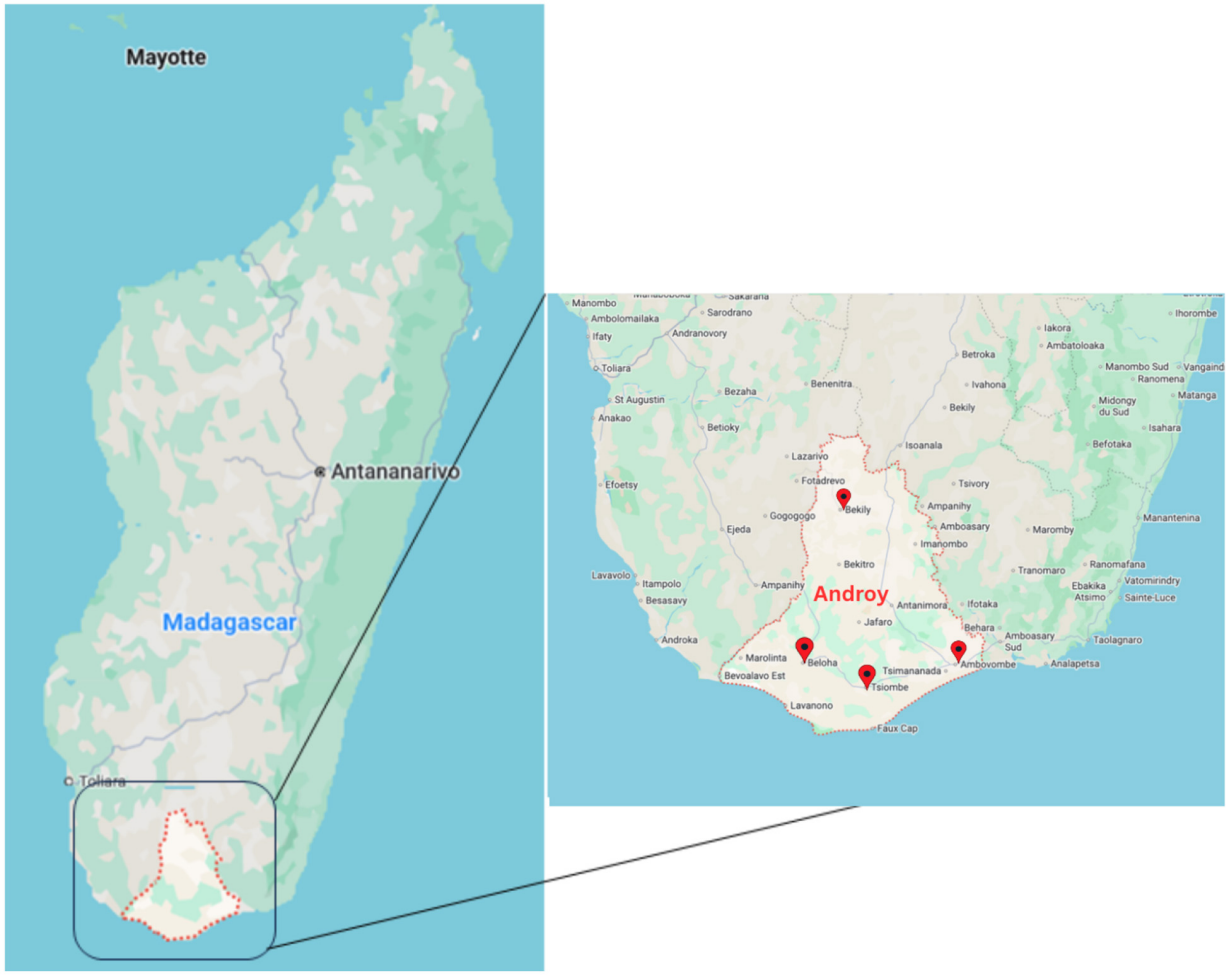
Map of Madagascar and Androy (22).

The fieldwork for this research was conducted in the Androy region of southern Madagascar, as illustrated in Figure 1.

This research was conducted by faculty and master's students from Central Androy Regional University (CURA) in collaboration with students from the Applied Global Public Health Initiative (AGPHI) at New York University's School of Global Public Health (NYU GPH). The study employed a cross-sectional mixed-methods design. This study was cleared ethically by the Androy region's Ministry of Higher Education and Scientific Research. Over the course of 2 months, from February 2024 to March 2024, CURA students collected data through focus group interviews and surveys administered to community members in the region, aiming to identify their main concerns related to climate change, the adaptation strategies that communities adopted, and the gaps in current approaches.

### 2.2 Recruitment & participants

Focus group participants were selected from the districts of Ambovombe, Androy, Tsihombe, Beloha, and Bekily. A total of 16 focus groups were conducted, comprising seven participants per group, three women and four men. Participants worked in various sectors related to agriculture, livestock, and fishing, and represented a range of educational levels, from illiterate to university degree holders. Traditional and church leaders were also included. Individuals under the age of 18 were excluded from participation. Informed consent was obtained verbally using the local dialect. A detailed description of each focus group is provided in Table 1.

**Table 1:** Focus groups description.

Focus group	Description
Focus group 1	Leader: Retired Gendarme Participants are fishers and have secondary education levels Participants from the southern coast, Faux Cap village, rural commune of Faux Cap
Focus group 2	Leader: Retired Teacher Participants are farmers and have secondary education levels Participants from Anja Tsihombe village, rural commune of Tsihombe, Tsihombe district
Focus group 3	Leader: Fisherman and Farmer. Participants are fishers and have primary education levels Participants from the southern tip of Madagascar's coast, in the village of Lavanono, in the rural commune of Tragnovaho, in the Beloha district
Focus group 4	Leader: Farmer and Village Chief Participants are farmers and have primary education levels Participants from Tragnovaho village, Tragnovaho rural commune, Beloha district
Focus group 5	Leader: Farmer Participants are farmers and have primary education levels Participants from Marovato rural commune of Marovato, Tsihombe district
Focus group 6	Leader: Retired Pastoralist and Farmer Participants are farmers and fishers and have secondary education levels Participants are from the Soamagnitse village, on the southern coast of Madagascar, in the rural commune of Tragnovaho, Beloha district
Focus group 7	Leader: Farmer and Village Chief. Participants are farmers and have primary education levels Participants are from Ankatrafae village, rural commune of Tragnovaho, Beloha district
Focus group 8	Leader: Farmer Participants are farmers and have primary education levels Participants are from Agnaretake village, Behazomanga rural commune, Beloha district
Focus group 9	Leader: Farmer and Village Chief Participants are farmers and have primary education levels Participants from Angodobo village, rural commune of Antanemora Sud, district of Ambovombe Androy
Focus group 10	Leader: Farmer and Village Chief Participants are farmers and have primary education levels Participants from Bemamba Tsarapioke village, rural commune of Antanemora Sud, district of Ambovombe Androy
Focus group 11	Leader: Farmer and Village Chief Participants are farmers and have primary education levels Participants from Tanantsoa village, rural commune of Antanemora Sud, district of Ambovombe Androy
Focus group 12	Leader: Farmer and Village Chief Participants are farmers and have primary education levels Participants from Mitsoriake village, rural commune of Ambohimalaza Sud, district of Ambovombe Androy, primary level.
Focus group 13	Leader: Mayor of the rural commune Participants are farmers and have primary education levels Participants from Bevitike village, rural commune of Bevitike South, district of Bekily
Focus group 14	Leader: Farmer and Village Chief Participants are farmers and have primary education levels Participants from Antsakoamaro village, rural commune of Antsakoamaro, district of Bekily, primary level
Focus group 15	Leader: Farmer and Village Chief Participants are farmers and have primary education levels Participants from the village Afondreambinta Vohidroe, rural commune of Belindo Mahasoa, district of Bekily
Focus group 16	Leader: Farmer and Village Chief Participants are farmers and have primary education levels Participants from Antsakoandahy village, rural commune of Belindo, district of Bekily

### 2.3 Focus group questions

During the interviews, the CURA team explained the study's purpose and advised on response confidentiality. Interview questions were consistent throughout the study using a survey sheet. Responses were tracked and stored using Excel and Microsoft Word, and team files were used for internal document sharing and repositories. The raw results are located in the Appendix.

Participants were asked various questions focused on the effects of climate change, such as: “Did you notice any changes in the climate compared to before the year 2000?” “What changes have you noticed and what are the consequences of these changes?” “What are the origins of the changes?” “What type of accommodations have you adopted in terms of livestock, fishing, health, water demand, forest conservation, schooling, income, and family?” They were also asked about adaptations made regarding migration and the effects of decisions made by political administration and humanitarian aid agencies. When the focus groups responded positively to the interview question, a value of 1 was assigned, and a value of 0 was assigned if the response was negative. The raw data with these values can be found in the Appendix.

### 2.4 Data analysis

To analyze the data from the focus group interviews, each question was assigned a value of either 1 or 0. A value of 1 indicates that a pattern was present based on the group's positive response, while a value of 0 signifies its absence. Python (14) was used for all the statistical analysis. Descriptive analysis was first conducted to develop a bar chart highlighting the 10 most prevalent themes among the focus groups, which allowed us to emphasize the issues and questions that received the highest number of affirmative responses. Additionally, in order to assess the similarities between focus groups, Dice-Sørensen coefficients were calculated. Indeed, these statistical measures are particularly appropriate for binary data, enabling cluster analysis of heterogeneous datasets. They allow the integration of qualitative data transformed into quantitative scores to identify co-occurrence across pairs. Given that the present study used a dataset based on the presence or absence of patterns, and this method is commonly used as a metric of association in co-occurrence analysis, the Dice–Sørensen coefficient was selected (15, 16). Dice-Sørensen coefficients were also employed to develop a Dice-Sørensen Similarity Heatmap and to perform hierarchical agglomerative clustering based on the similarity matrix. Similarity values were converted to distances, and clustering was conducted using average linkage (UPGMA), where cluster similarity is defined as the average distance between all pairs of observations within a cluster. This approach is commonly used for presence-absence data and supports the identification of groups sharing similar thematic patterns (15–17).

## 3 Results

**Figure 2:**
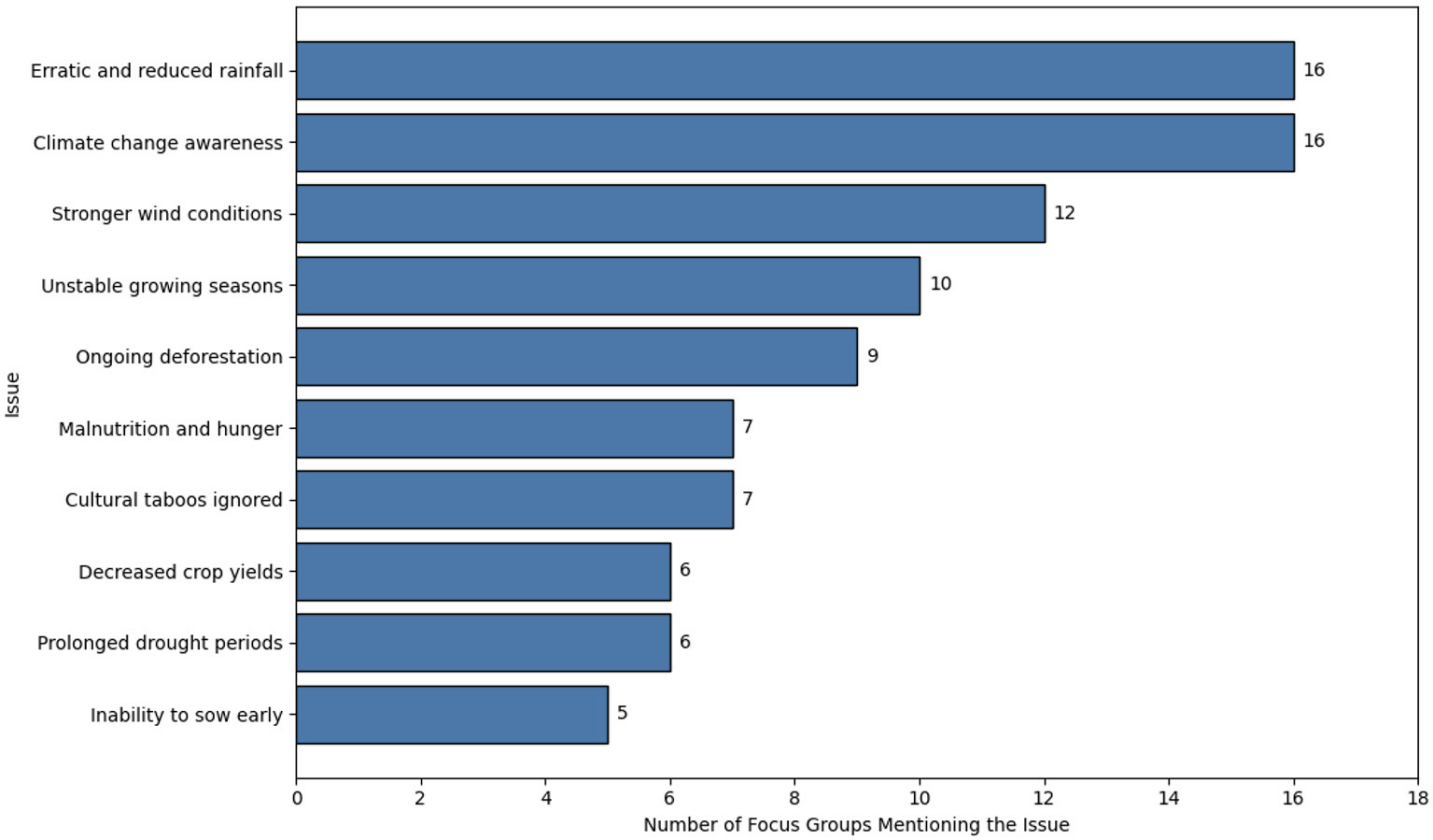
Theme prevalence chart in the focus groups.

The majority of participants came from farmer groups in rural communities, with a smaller portion representing fishing groups. Focus group leaders were typically farmers, village chiefs, teachers, or gendarmes. Differences were primarily informed by geographic location. Participants were included from multiple rural districts, including Faux Cap village, Anja Tsihombe, Angobodo, and Antsakoamaro. To examine the main issues faced by the focus groups from these localizations experiencing climate change, responses were analyzed in percentage terms to assess their frequency concerning specific questions. Figure 2 presents a bar chart illustrating the most frequent issues across all focus groups.

This illustrates the 10 challenges most frequently encountered by the community, showcasing the main stressors impacting resilience in the Androy region. Among the three key themes depicted in the frequency chart, issues including unstable growing seasons, erratic or reduced rainfall, and awareness of climate change comprise 11.5% of the 10 questions.

**Figure 3:**
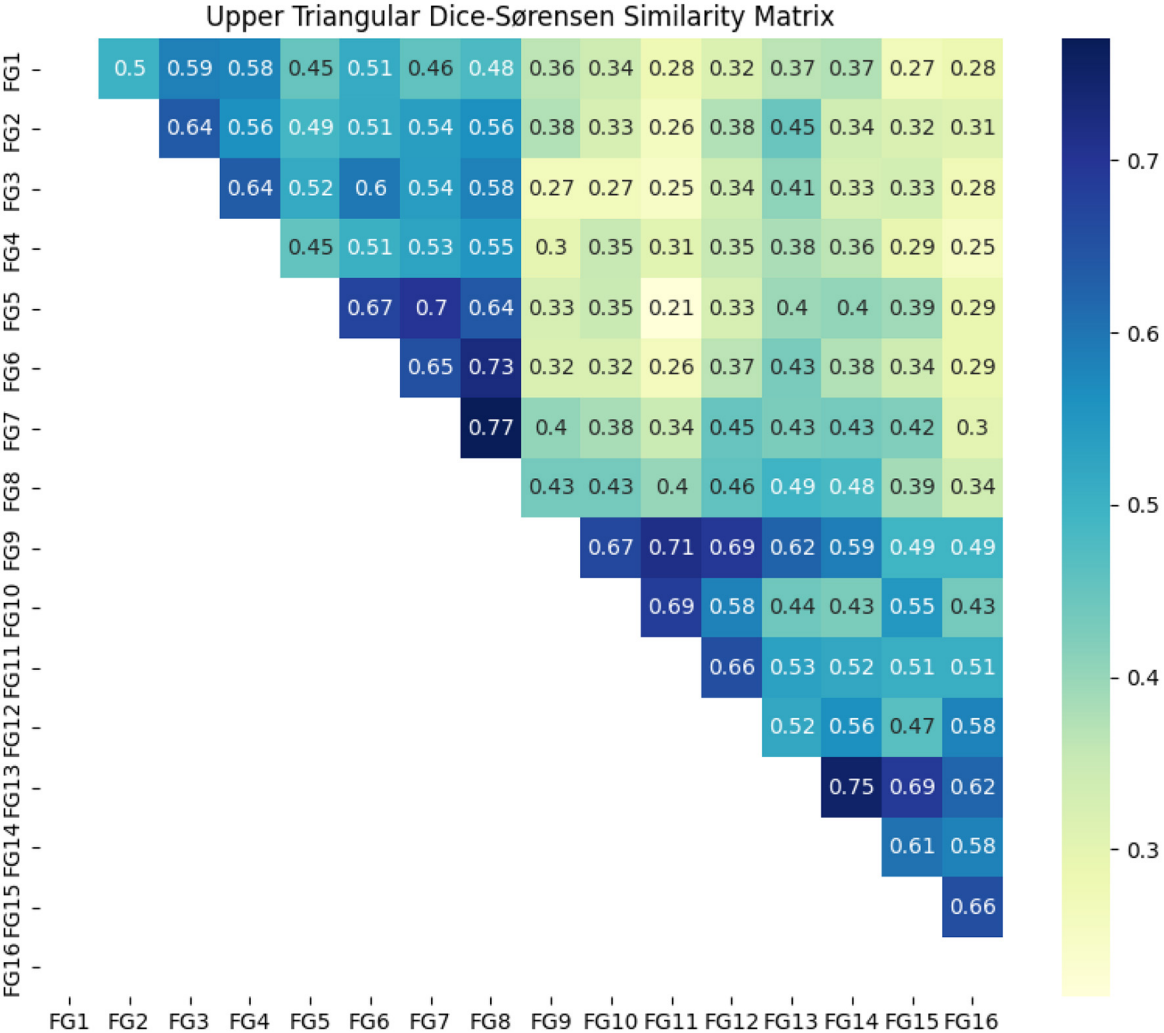
Dice-Sørensen similarity heatmap.

To assess the similarities and differences observed during the interviews within the focus groups, the Dice-Sørensen coefficient was calculated to generate, first, a Dice-Sørensen similarity heatmap. This heatmap, illustrated in Figure 3, aids in identifying high-similarity clusters. As the similarities among focus groups increase, the coefficient in the matrix approaches 1.

**Figure 4:**
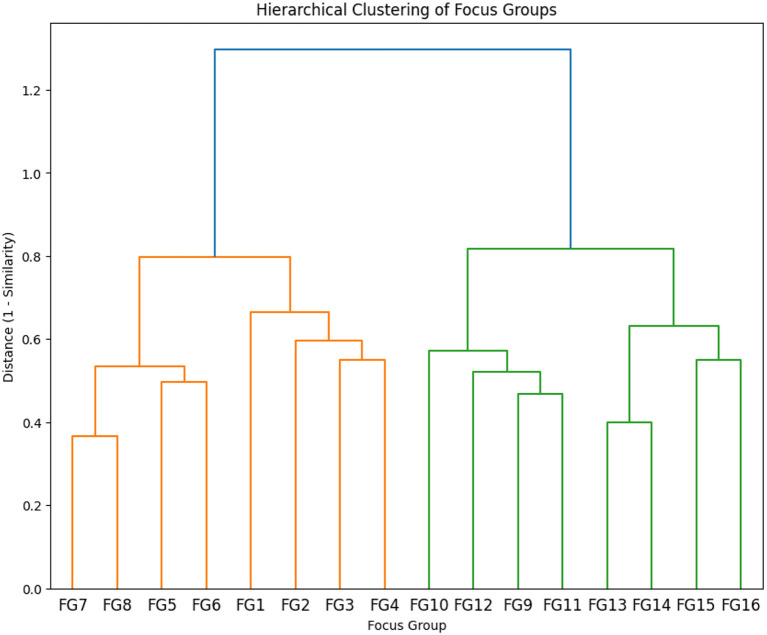
Hierarchical clustering of focus groups based on Dice–Sørensen similarity.

The focus groups were categorized into high-similarity clusters based on Dice-Sørensen similarity coefficients. These coefficients enabled us to create a dendrogram, as shown in Figure 4, where the cluster analysis provides a visualization of the hierarchical clustering of focus groups, illustrating the relationships and similarities between different groups.

The dendrogram shows two main branches. The first branch includes focus groups from Faux Cap, Tsihombe, and Beloha, whereas the second branch comprises groups from Ambovombe Androy and Bekily (Table 1), indicating alignment with district-level geographic distribution.

Groups that merge lower on the dendrogram tend to share more similar themes. For example, FG7, FG8, FG5, and FG6 form a cluster on the left part of the dendrogram, indicating relatively high similarity in their themes.

The key themes for each cluster are extracted and displayed in Table 2.

**Table 2:** Themes for high-similarity clusters.

Cluster	Focus groups	Prevalent themes	Interpretation
Cluster 1	FG1, FG2, FG3, FG4	Community-driven reforestation, healthcare needs, and humanitarian agri i'm cultural dependence	Efforts must be invested in supporting reforestation, agriculture, and economic resilience.
Cluster 2	FG5, FG6, FG7, FG8	Health challenges, reliance on humanitarian aid, seed multiplication, and family labor	Highlighting economic vulnerability and community-based adaptations
Cluster 3	FG9, FG10, FG11, FG12	Disease prevalence, limited healthcare, and family work	Focus on health resilience and shared economic challenges
Cluster 4	FG13, FG14	Water scarcity, educational needs, and reforestation	Community resilience through environmental and educational efforts

Four clusters with highly similar patterns were found in this analysis, the corresponding focus groups consisted of a mix of fishers and farmers with FG 1, 2, and 6 having secondary education levels while the remaining groups had primary education levels. The corresponding focus groups also consisted of participants from each of the study districts. During the interviews, it was observed that all the clusters were experiencing various climate impacts, such as rain irregularities or disorganized growing seasons, as well as water scarcity. Each cluster shows the prevalent themes coming from the focus group answers about their main concerns, their strategies adopted, the gaps to resilience, and the solutions suggested.

FG15 and FG16, both consisting of farmers with primary education levels from the Bekily district, have a low score of similarities with the other groups and present key themes including specific climate impacts, health and water challenges, unique agricultural practices, and distinct educational needs. Table 3 below shows the unique pattern in these two groups, emphasizing specific needs and adaptation strategies unique to FG15 and FG16.

**Table 3:** FG15 and FG16 groups results.

Theme	Similarities between FG15 and FG16	Unique groups (FG15 and FG16)
Climate and environment	Erratic rainfall, deforestation	Reforestation (FG15), water scarcity adaptations (FG16)
Healthcare and disease	Disease prevalence, vaccination reliance	Healthcare infrastructure (FG15)
Economic resilience	Family labor, income diversification	Climate-resistant crops (FG16), adapting outdated practices (FG15)
Education and infrastructure	Qualified teachers, water pipelines	Nearby healthcare facilities (FG15), skilled teachers (FG16)

According to Table 3, FG15 highlighted the need for healthcare infrastructure and revealed a strong reliance on outdated agricultural practices. Participants in FG16 emphasized the importance of education in strengthening resilience and reported experimenting with climate-resistant crops.

## 4 Discussion

This study aims to identify the major issues encountered by communities in the Androy region of Madagascar by examining the impact of climate change. Figure 2 illustrates the 10 major issues present in the interview answers from the focus groups. The predominant concerns were erratic or reduced rainfall and general climate change awareness. Following these, stronger wind conditions, prolonged droughts accounted, stronger winds, and difficulties with early sowing and reduced crop yields for, indicating a high risk of crop failure in their localizations. Additionally, a loss of traditional cultural taboos was noted, along with challenges related to deforestation, malnutrition, and hunger. Cultural taboos significantly impact Malagasy society, often influencing natural resource usage by safeguarding certain species and habitats (3). These results indicate that the loss of cultural traditions could potentially harm resilience.

Linking these results with the demographics in Table 1 indicates that farmers and fishermen from rural regions in Androy recognize climate change and stress the importance of developing community-based resilience strategies and participatory planning in areas significantly affected by malnutrition and hunger. The agricultural challenges and climate change issues identified reinforce the urgent need for appropriate agricultural methods, especially to address extended droughts, erratic rainfall, and deforestation. The breakdown of cultural taboos that once protected biodiversity offers an opportunity for resilience and could address the erosion of cultural heritage. The connection between malnutrition and hunger, along with deforestation, highlights how forest loss further exacerbates these issues. Findings show that deforestation correlates with poorer nutritional outcomes, with agricultural expansion being a key factor in forest depletion (4). These points are critical to consider in mitigating climate-related food insecurity in Androy.

To evaluate the connection between focus groups and to determine any relationships among the concerns, adaptations developed, identified gaps, and the proposed solutions from the focus groups, a Dice-Sørensen coefficient analysis was employed. The findings from the Dice-Sørensen analysis revealed four distinct clusters and two focus groups that did not achieve a sufficient score to form a cluster. This analysis recognized four clusters with notably similar patterns. Cluster 1 included the first four focus groups and highlighted common challenges faced by the communities, such as varying climate impacts like inconsistent rainfall, disrupted growing seasons, and water shortages. To address these issues, community-based reforestation efforts were initiated. Additionally, this cluster highlighted resilience gaps in healthcare, such as high treatment costs and insufficient skills among healthcare providers. Moreover, a major obstacle affecting the resilience of these communities is their reliance on humanitarian aid, which is often limited and appears inadequately tailored to their needs.

Cluster 2 faces climate challenges similar to those of Cluster 1, though trends differ in terms of adaptation strategies and gaps observed by the focus groups. This highlights the impossibility of early sowing, leading to a disordered agricultural season, compounded by periodic dryness, which results in direct health consequences of malnutrition, the emergence of new diseases, and the spread of existing ones. As an adaptive strategy, communities have focused on increasing sowing, implementing short agricultural cycles, and planting resistant crops. In Cluster 2, reforestation, income diversification, and food crop promotion have helped boost monthly incomes. Education access improvements have been made by prohibiting child marriage and increasing school enrollment, while many families have become more involved in labor activities. However, challenges persist, such as seed distribution delays post-rainy season, insecurity in livestock breeding, high healthcare costs, and limited water access. Inadequate enforcement of forest conservation laws and educational infrastructure remains inadequate. Humanitarian aid is often perceived as a selective and non-viable solution, as many are left in need, which diminishes its impact and credibility. Suggested solutions include timely livestock vaccinations and constructing schools staffed with qualified teachers.

Cluster 3 experiences climate-related issues similar to Clusters 1 and 2. Their unique strategies for resilience involve traditional breeding, community-driven reforestation, and migration north to Tolaria in search of work. They believe that government solutions for agriculture are insufficient, citing a lack of control over livestock sale prices, insufficient irrigation water, and poverty resulting in the sale of wood. Moreover, there is an ongoing absence of teachers, leaving children without access to education. A lack of government support and insufficient humanitarian aid selectively provided to certain populations leads to increased migration. Suggested solutions include training agricultural workers, providing vaccinations for livestock, and constructing water pumps.

Cluster 4 exhibits similarities with the other clusters. A notable issue is the irregular sowing that follows rain, resulting in decreased zebu populations due to limited natural resources. FG15 emphasized the importance of agricultural practices and adaptations of traditional methods, showcasing how these management strategies address related challenges shown in Table 2. This indicates a need to improve productivity while navigating current difficulties and to enhance resilience against climate variability. FG16 points out that education and agriculture are crucial for fostering long-term resilience. These findings highlight community resilience and suggest that more focus is needed on education and accessible local health services.

The cluster analysis revealed resilience strategies underlining context-specific priorities in the Androy region. While all four clusters recognized the effects of irregular rainfall and prolonged droughts, their adaptive measures differed considerably, highlighting the difficulty of responding effectively to communities facing climate change. Clusters 1 and 2 focused on community-led reforestation, with Cluster 2 additionally implementing short-cycle agriculture, resilient crops, and educational initiatives, including banning child marriage and boosting school enrollment. Regarding Cluster 3, one specific challenge was the labor migration to Toliara, along with a critique of inadequate state regulation regarding livestock markets and irrigation access, revealing economic and governance shortcomings. Conversely, Cluster 4 focused on integrating traditional agricultural practices and the importance of education for long-term resilience.

The analysis of focus groups reveals that rural communities in Androy, including farmers and fishers, have developed strategies to combat climate change that require better support. These strategies emphasize community-led reforestation, short-cycle agriculture, resistant crops, and educational initiatives. Moreover, in terms of healthcare, access to medical services and the lack of skills among the healthcare workforce were the main gaps highlighted by some clusters. Furthermore, investments in environmental protection and social cohesion could be enhanced by promoting cultural taboos through their integration into rural resilience planning. Finally, humanitarian aid has been noted as being too selective regarding nutritional foods, leading to a high reliance on them. This reliance should also be considered to promote resilience strategies that enable long-term adaptation to climate change and reduce migration in these communities.

### 4.1 Gaps in current adaptation strategies & implications for policy and practice

Madagascar's government has enacted policies and governance measures to enhance climate responses, such as promoting renewable energy, reforestation, and climate-smart rice farming. The government is also developing systems integrating community support with resilient agricultural models. While political commitment exists for effectively implementing climate change adaptation strategies, significant challenges remain, including inadequate climate finance and insufficient infrastructure to address climate-related risks. According to the World Bank, its National Adaptation Program must invest in specific resilience adaptation (4).

Despite ongoing efforts to adapt to climate change, southern Madagascar remains structurally dependent on humanitarian aid to meet basic needs. While aid addresses immediate crises, its discontinuity often hinders the development of sustainable systems, making communities vulnerable when support fluctuates. This dependency highlights capacity gaps between different localities, both in terms of limited access to timely climate data and early warning systems, and differences between urban and rural areas. Without proactive climate information, communities are forced to apply reactive adaptation measures. To increase resilience, it is critical to establish local information centers and empower communities to use climate data.

Moreover, inadequate investment in sustainable health infrastructure, especially since water management hinders resilience efforts. While some areas have benefited from pilot projects on water-saving management and efficient irrigation, the absence of region-wide approaches leaves many communities suffering from drought. Furthermore, educational gaps restrict the community's ability to implement advanced agricultural technologies. Improving educational resources and providing training in sustainable agriculture will also allow communities to play a more active role in building long-term resilience.

### 4.2 Comparison with other regions or studies

Southern Madagascar's adaptation efforts reveal the common approaches compared to other climate-affected regions. The impacts of drought and unpredictable weather patterns have prompted communities to adopt measures such as short-rotation cropping and community-led reforestation (18). At the same time, a reliance on humanitarian assistance and traditional agricultural practices reflects a widespread need for immediate and accessible (18).

In other areas, such as the Atsinanana and Analamanga regions, communities have adopted similar adaptation strategies but benefited from additional support structures (18). For example, the United Nations Development Programme (UNDP) project in these areas provides access to weather and climate information, enabling local communities to make informed decisions about when to plant crops and manage water resources. In contrast, the Androy region's lack of basic infrastructure restricts its access to timely data and more reactive methods (19). Poor infrastructure in roads and limited market access greatly impact farmers' income and the community members that rely on local produce and markets.

Furthermore, adaptation also includes innovative water-saving technologies, such as drop-by-drop irrigation systems and solar-powered water pumps, supported by organizations like UNICEF and WFP. These interventions have helped stabilize crop yields and offer a model for drought resilience. In other high-risk areas, integrated systems like early warning mechanisms are emphasized to strengthen adaptive responses. Through the UN's Early Warnings for All, these approaches promote proactive actions to help vulnerable communities reduce risk and build up resilience against future climate challenges (20). Other regions in Madagascar have shown strategies such as changing the timing of crop planting to better fit changing rainfall patterns, diversifying crops, and temporary migration depending on the season to earn additional income (21).

### 4.3 Limitations of the study & future research directions

Several limitations should be taken into account when interpreting the findings. Firstly, this method could introduce information bias since the qualitative data is derived from interviews. Responses from focus groups may not adequately capture the range of experiences across all communities in southern Madagascar, as some viewpoints might be over- or under-represented. Notably, the communities involved in the focus groups predominantly comprise farmers and fishers from rural areas, which could signify a selection bias.

Additionally, this study examines specific adaptation strategies in the Androy region, which may vary from those employed in other parts of Madagascar that face different climate challenges. The interview questions posed were closed-ended and did not allow focus group participants to elaborate on additional aspects.

These issues may constrain the applicability of the study's findings on a broader scale. Future research could investigate community adaptations in both urban and rural settings, as well as the long-term effectiveness of specific adaptation measures identified in this study within the Androy region, utilizing longitudinal data to track changes over time. Potential areas of exploration could include targeted interventions like the effects of early warning systems, enhanced local education initiatives, and improved water management practices. Another focus could be the evaluation of local governance and policy support's role in bolstering resilience, offering insights on how to integrate external assistance with community-led efforts. Although centered on Androy, the insights gleaned from this study could provide valuable advice for policymakers and local governance aiming to bolster community resilience strategies.

## 5 Conclusion

This study examined the climate change challenges faced by communities in the Androy region, located in southern Madagascar, along with the resilience strategies developed in response. We used a mixed-methods design that included focus group interviews with participants primarily from the rural farming and fishing sectors. Significant issues were identified, including unstable growing seasons, unpredictable rainfall, extended droughts, and food insecurity. Additionally, communities reported a decline in traditional environmental practices and limited access to health and education services.

By applying the Dice–Sørensen similarity coefficient, four thematic clusters of communities were identified to have shared concerns and adaptive strategies such as community-led reforestation, short-cycle agriculture, and educational initiatives. Nonetheless, persistent gaps were noted, including insufficient government support, selective humanitarian aid, and poor infrastructure.

These findings underscore the pressing need for tailored, community-driven interventions that leverage local knowledge and enhance essential services to foster long-term resilience against climate change.

## Data Availability

The raw data supporting the conclusions of this article will be made available by the authors, without undue reservation.
