# Supplementary material for: Assessing vulnerabilities and resilience strategies for communities facing climate change in Androy, Southern Madagascar
Source: Front Public Health. 2026 Apr 9;14:1747679. doi: 10.3389/fpubh.2026.1747679 (PMC13116547; doi:10.3389/fpubh.2026.1747679)
Supplement: Supplementary file 1 [file Data_Sheet_1.pdf]

Appendix

FOCUS GROUPE DANS LA REGION ANDROY

N°	Questions	Questions	FG1	FG2	FG3	FG4	FG5	FG6	FG7	FG8	FG9	FG10	FG11	FG12	FG13	FG14	FG15	FG16	Total points
1	**Climate Change**	**Question Changement climatique**																	
2	**Did you feel a change in climate compared to the year or before 2000?**	Avez-vous sentir un changement climatique par rapport à l'année ou avant 2000?	1	1	1	1	1	1	1	1	1	1	1	1	1	1	1	1	**16**
3	**What have you noticed?**	**Qu'est-ce que vous avez remarqué ?**																	**0**
4	Erratic rainfall	Irrégularité des pluies	1	1	1	1	1	1	1	1	1	1	1	1	1	1	1	1	**16**
5	Advancement of large-area dunes	Avancement des dunes à grande surface	1																**1**
6	Disorganized growing season	Saison culturale désorganisée	1	1	1	1	1	1	1	1	1	1							**10**
7	Irregularity and inadequacy of rainfall	Irrégularité et insuffisance des pluies	1		1	1	1	1		1					1	1			**8**
8	Disappearance of forests	Disparition des forêts	1				1												**2**
9	Firewood problem	Problème de bois de chauffe	1																**1**
10	Malnourished child	Enfant malnutris		1															**1**
11	Increased wind intensity	Augmentation de l'intensité du vent		1	1				1	1	1	1	1	1	1	1	1	1	**12**
12	Water Problem Amplification	Amplification de problème d'eau					1												**1**
13	Multiplication of diseases	Multiplication des maladies,		1															**1**
14	Diabetes	Diabète,		1															**1**
15	Fibroma	Fibrome		1															**1**
16	Malaria	Paludisme		1															**1**
17	Appendicitis	Appendicite		1	1														**2**
18	COVID-19	COVID 19		1															**1**
19	Hepertension	Hypertension		1															**1**
20	Early sowing impossible	Semis précoce impossible			1		1	1	1	1									**5**
21	Reduction in agricultural production	Réduction de la production agricole			1	1			1	1				1		1			**6**
22	Multiplication of the area occupied by grasshoppers	Multiplication de surface occupé par les sauterelles			1														**1**
23	Surrounding temperature increase	Augmentation de température environnante			1								1						**2**
24	**If there are any changes; In your opinion, what are the origins of the change?**	**S'il y a des changements ; selon vous quels sont les origines du changement ?**																	**0**
25	Use of chemicals	Utilisation des produits chimiques	1												1	1	1	1	**5**
26	Non-respect of taboo	Non-respect de tabou	1	1	1	1	1		1	1									**7**
27	Destruction of primary forest	Destruction de la forêt primaire		1		1	1	1	1	1	1	1		1					**9**
28	Wildland fire	Feu de végétation											1						**1**
29	**What are the consequences of these changes?**	**Quels sont les conséquences de ces changements?**																	**0**
30	Reducing the lifespan of human beings	Réduction de la durée de vie des êtres humains	1																**1**
31	Malnutrition	Malnutrition,		1	1		1	1	1						1		1		**7**
32	Multiplication of diseases	Multiplication des maladies		1	1		1	1	1	1				1	1		1		**9**
33	Disorganized growing season	Saison culturale désorganisée		1	1		1	1	1	1				1					**7**
34	Low purchasing power	Faible pouvoir d'achat			1												1		**2**
35	Diarrhea and frequent malnutrition	Diarrhée et malnutrition fréquent	1																**1**
36	Temperature increase	Augmentation de températures					1								1	1			**3**
37	Periodic drought	Sécheresse périodique					1	1	1	1	1	1							**6**
38	Erratic rainfall	Irrégularité des pluies						1		1					1	1			**4**
39	Reduced soil fertility	Réduction de la fertilité du sol									1		1		1	1			**4**
40	Proliferation of caterpillars and insect pest	Prolifération des chenilles et insectes nuisibles							1										**1**
41	Reduction of the day of the sea trip, for fishing, due to the presence of wind	Réduction de jour de la sortie en mer, pour pêche, suite à la fréquentation de vent	1		1														**2**
42	**What types of accommodations have you adopted?**	**Quels sont les types d'adaptations que vous avez adoptées?**																	**0**
43	***At the level of agriculture***	***Au niveau de l'agriculture***																	**0**
44	All-weather sowing after rains	Semis tout temps après la pluies	1				1				1	1	1	1					**6**
45	Use of local seeds	Utilisation de semences locales													1		1		**2**
46	Use of chemicals to treat production insect pests	Utilisation des produits chimiques pour traiter les insectes ravageurs de production		1															**1**
47	Seedling and seed multiplication	Multiplication de semis et de semences			1		1	1	1	1									**5**
48	Short-cycle agriculture	Agriculture a cycle court					1	1	1	1									**4**
49	Planting of resistant varieties	Plantation de variété résistante					1	1	1	1						1			**5**
50	Small-scale irrigation,	Petite irrigation,							1	1									**2**
51	Application of Agriculture Training	Application de la formation sur l'agriculture																1	**1**
52	Use of the veterinarian to treat caterpillars	Recours au vétérinaire pour traiter les chenilles				1													**1**
53	Use of water-efficient technology	Utilisation de technique à consommation d'eau réduite							1	1									**2**
54	Use of medicinal plants to control caterpillars.	Utilisation des plantes medécinale pour maîtriser les chenilles.	1			1													**2**
55	Clearing of primary forest that is productiv	Défrichement de forêt primaire qui sont productives,								1									**1**
56	***At the livestock level***	***Au niveau de l'élevage***																	**0**
57	Mastering Insecurity from Tradition	Maitrise de l'Insécurité à partir de la tradition	1								1	1							**3**
58	Autoveterinary	Autovétérinaire		1															**1**
59	Multiplication of diseases	Multiplication des maladies			1		1	1		1									**4**
60	Stop of transhumance	Arrêt de la transhumance				1													**1**
61	Resistivity of diseases against drugs	Résistivité des maladies contre les médicaments					1		1	1									**3**
62	Insufficient medication	Médicament insuffisant						1											**1**
63	Number of herds significantly reduced	Nombre de cheptel significativement réduit					1												**1**
64	Zebu wandering in the forest	Divagation de zébus dans la forêt									1		1	1	1	1		1	**6**
65	Cactus Pastoralism	Pastoralisme de cactus										1					1		**2**
66	***In terms of fishing***	***Au niveau de la pêche***																	**0**
67	Offshore fishing and the proliferation of fis	Pêche au large et multiplication des engins de pêche	1		1			1											**3**
68	The coastal community became a fish consumer and fisherman	La communauté du littorale devenue consommateur de poissons et pêcheur		1				1											**2**
69	***At the health level***	***Au niveau sanitaire***																	**0**
70	Hospital attendance closer to the village	Fréquentation des hôpitaux plus proche du village	1	1														1	**3**
71	NGO support for children and pregnant wo	Appui des ONG aux enfants et des femmes enceintes	1		1														**2**
72	Use of moderately vitamin-rich food as possible	Utilisation de nourriture moyennement riche en vitamine suivant la possibilité	1																**1**
73	Burning Croton ssp *plants* to keep the Malaria-carrying Mosquito away	Brûlure des plantes *Croton ssp* pour éloigner le Moustique porteur de Malaria			1														**1**

74	Use of medicinal plants	Utilisation de plantes médicinales				1			1			1	1		1	1	1		**7**
75	Appearance of new disease (Appendicitis, Fibroid, Cyst, Coronavirus,)	Apparition de nouvelle maladie (Appendicite, Fibrome, Kyste, coronavirus,)					1	1	1	1									**4**
76	Increase in self-physician/self-treatment	Augmentation des automedecin/autotraitement							1		1			1					**3**
77	The cost of treatment is not within the reach of patients.	Le cout de traitement n'a pas à la portée des patients.							1	1									**2**
78	Sale of medication at the grocery store level	Vente de médicament au niveau des épiceries				1													**1**
79	***When needed for water***	***Au besoin d'eau***																	**0**
80	There remains the problem of the Androy	Reste problème de l'Androy	1	1	1			1	1	1					1			1	**8**
81	Reduction of daily water consumption	Réduction de consommation journalière de l'eau				1													**1**
82	Use of unclean pond water	Utilisation de l'eau de mare non propre						1	1	1		1	1	1		1	1		**8**
83	***Forest Conservation***	***Conservation de la forêt***																	**0**
84	Reforestation	Reboisement	1	1	1	1	1	1	1	1					1	1	1	1	**12**
85	Establishment of forest managed by the grassroots community	Mise en place de forêt gérer par la communauté de base					1	1	1	1	1	1	1	1	1	1	1	1	**12**
86	***Schooling***	***Scolarisation***																	**0**
87	Schooling of children	Scolarisation des enfants	1	1			1	1	1	1	1			1	1	1		1	**11**
88	Prohibition of early marriage	Interdiction du mariage précoce	1				1	1	1	1						1	1		**7**
89	Constriction of schools, even straw, by vill	Constriction des écoles, même en paille, par village				1	1	1	1	1		1	1						**7**
90	***Monthly income***	***Revenu mensuelle***																	**0**
91	Multiplication of income-generating sub-a	Multiplication des sous activités génératrices de revenu	1	1	1	1	1	1	1	1		1							**9**
92	Promotion of food crops	Promotion de culture vivrière					1	1	1	1	1		1	1	1	1	1	1	**11**
93	Promotion of small-scale fisheries	Promotion de la pêche artisanale						1											**1**
94	***At the family level***	***Au niveau familial***																	**0**
95	Involvement of family members in daily activities	Implication des membres de famille aux activités journalières	1	1	1	1	1	1	1	1		1		1	1	1	1	1	**14**
96	Multiplication of activities	Multiplication des activités						1		1	1		1		1		1		**6**
97	***Migration***	***Migration***																	**0**
98	Move to the North of Madagascar, to Toliara, and other productive localities in search of sustainable employment	Déplacement vers le Nord de Madagascar, à Toliara, et autres localité productives à la recherche de l'emploi durable	1	1	1	1	1		1	1	1	1	1		1	1	1		**13**
99	Temporary migration at the regional level	Migration temporaire au niveau de la région						1						1				1	**3**
100	***Respect for taboos***	***Respect de tabou***																	**0**
101	Abandonment of certain traditions that hav	Abandon de certaines traditions dépassées par le temps	1	1	1	1	1	1	1	1	1	1	1	1	1	1	1	1	**16**
102	**What do you think are the gaps in resilie**	**Selon vous quels sont les lacunes de la résilience**																	**0**
103	***At the level of agriculture***	***Au niveau de l'agriculture***																	**0**
104	Non-control of insects and caterpillar pests of the crop	Non maîtrise d'insectes et chenille ravageurs de la culture	1													1			**2**
105	Distribution of seeds after the rainy season	Distribution de semences après la saison des pluies.	1				1	1	1	1									**5**
106	Population dependence on humanitarian ai	Dépendance de population aux aides humanitaires,		1	1	1		1		1					1				**6**
107	Inadequate solution	Solution non adéquat									1	1	1	1			1	1	**6**
108	***At the livestock level***	***Au niveau de l'élevage***																	**0**
109	Vaccine and ineffective treatment,	Vaccin et traitement inefficace,	1		1	1		1		1									**5**
110	Insecurity	Insécurité			1	1	1	1	1					1	1	1	1	1	**10**
111	Non-control of the selling price of livestoc	Non maitrise de prix de vente des cheptels									1	1	1		1				**4**
112	Problem of grazing land due to scarcity of rainfall	Problème de terrain de pâturage suite à la rareté des pluies															1	1	**2**
113	The hens distributed by NGOs are not effective, dead after distribution	Les poules distribuées par les ONG ne sont pas efficace, morts après distribution					1												**1**
114	***In terms of fishing***	***Au niveau de la pêche***																	**0**
115	Lack of fishing equipment	Insuffisance de matériels de pêche	1		1			1											**3**
116	the fishing zone has become further and fu	la zone de pêche devenue plus en plus loin de la côte.	1		1														**2**
117	***At the health level***	***Au niveau sanitaire***																	**0**
118	very expensive treatment cost compared to the communities	coût de traitement très cher par rapport au moyen des communautés	1	1			1	1	1	1	1				1	1	1		**10**
119	Moderately qualified and unequipped doct	Médecin moyennement qualifié et non équipé	1		1	1		1											**4**
120	Basic health far from other villages.	Santé de base loin des autres villages.						1											**1**
121	Lack of equipment	Insuffisance de matériels						1											**1**
122	Ban on medicinal plants	Interdiction de plantes médicinales								1		1	1	1				1	**5**
123	***When needed for water***	***Au besoin d'eau***																	**0**
124	Lack of water	Manque d'eau	1	1	1	1	1	1	1	1					1	1	1	1	**12**
125	Lack of water for irrigation	Absence d'eau pour irrigation									1	1	1	1					**4**
126	***Forest Conservation***	***Conservation de la forêt***																	**0**
127	Non-application of current law	Non application de loi en vigueur	1	1	1	1	1	1	1	1									**8**
128	Lack of financial and material resources	Manque de moyen financier et matériel						1											**1**
129	Acute poverty leads to the sale of wood	La pauvreté aiguë entraine la vente de bois									1	1	1	1	1	1	1	1	**8**
130	***Schooling***	***Scolarisation***																	**0**
131	Lack of adequate infrastructure	Insuffisance des infrastructures adéquates	1	1	1	1	1	1	1	1									**8**
132	Low level of students and teachers	Bas niveau des élèves et des enseignants	1	1	1	1			1										**5**
133	No inspection on education	Pas d'inspection sur l'éducation							1										**1**
134	Continuous absence of teachers, a source of abandonment of children.	Absence continue des Enseignants, source d'abandon des enfants.								1	1	1	1	1	1	1	1	1	**9**
135	Population poverty	Pauvreté de la population													1			1	**2**
136	***Monthly income***	***Revenu mensuelle***																	**0**
137	Lack of IGA, non-industrialization	Manque d'AGR, non industrialisation	1	1	1	1		1	1	1	1	1	1	1					**11**
138	Dependence on agriculture	Dépendance à l'agriculture													1	1	1	1	**4**
139	***Women and children***	***Les femmes et des enfants***																	**0**
140	Early sexual act of some girls following poverty	Acte sexuel précoce de certaines filles suite à la pauvreté								1		1							**2**
141	Dependence of children and women on the father of the family	Dépendance des enfants et femmes au père de famille									1				1	1	1	1	**5**
142	Migration to the big city	Migration vers la grande ville											1						**1**
143	Grocery store creation	création épicerie												1					**1**
144	***Decision of the administration***	***Décision de l'administration***																	**0**
145	Sometimes a decision has a political tender	Parfois une décision a tendance politique	1																**1**
146	Selective humanitarian aid, cannot reach the general population	Aide humanitaire sélective, ne peut pas toucher la population en générale		1	1	1	1	1	1	1									**7**

147	Non-responsible administration	Administration non responsable									1	1	1	1	1	1	1	1	**8**
148	***Assistance from the Malagasy State***	***Assistance de l'état Malagasy***																	**0**
149	Moderately visible and affects only a few populations	Moyennement visible et ne touche que quelque population	1	1				1		1	1	1	1	1	1	1			**10**
150	Humanitarian aid promotes famine.	L'aide humanitaire favorise la famine.							1										**1**
151	Inadequate solution	Solution non adéquat					1										1	1	**3**
152	***At the level of humanitarian aid agencies***	***Au niveau des organismes d'aide humanitaire***																	**0**
153	Distribution of living just selective.	Distribution de vivre juste sélective.	1	1	1	1				1	1	1	1	1	1	1	1	1	**13**
154	Humanitarian aid is not a solution.	L'aide humanitaire n'est pas une solution.					1	1	1										**3**
155	***At the level of individuals.***	***Au niveau des individus.***																	**0**
156	Permanent or temporary migration,	Migration définitive ou temporaire,	1						1		1	1	1	1					**6**
157	Dependence on humanitarian aid	Dépendance aux aides humanitaires		1	1	1		1		1									**5**
158	Only the disabled, pregnant and breastfeeding who enjoyed the aids	Seule les handicapé, enceinte et fait allaiter qui jouirent les aides					1												**1**
159	**Proposal of solutions**	**Proposition des solutions**																	**0**
160	***Fishing***	***Pêche***																	**0**
161	Provision and use of speedboats and training of fishermen	Dotation et utilisation de vedette et formation des pêcheurs	1		1			1											**3**
162	***Agriculture***	***Agriculture***																	**0**
163	Timely distribution of humanitarian aid,	Distribution en temps des aides humanitaire,	1			1													**2**
164	Establishment of basic infrastructure,	Mise en place des infrastructures de base,	1			1		1	1	1			1						**6**
165	Insect Pest Treatment	Traitement des insectes nuisible	1			1								1					**3**
166	Crop land irrigation	Irrigation de terrain de culture								1									**1**
167	Everyone must do what is necessary to deal with the famine	Chacun doit faire le nécessaire pour faire face à la famine		1	1		1												**3**
168	Farmer training	Formation des agriculteurs									1	1			1		1		**4**
169	Food Crop	Culture vivrière														1			**1**
170	Suitable seed	Semence adaptée																1	**1**
171	***Breeding***	***Elevage***																	**0**
172	Vaccine in time,	Vaccin en temps,	1	1	1	1	1	1	1	1	1	1	1	1	1		1	1	**15**
173	Control of insecurity	Maitrise de l'insécurité	1				1	1				1							**4**
174	Setting up of Veterinarians by village grou	Mise en place de Vétérinaire par groupe de village									1					1			**2**
175	Setting up Impluvium by village group	Mise en place de Impluvium par groupe de village												1					**1**
176	***Water***	***Eau***																	**0**
177	Construction of functional pipelines for agriculture and humans	Construction de pipeline fonctionnelle pour agriculture et les humains	1	1	1	1	1	1											**6**
178	Installation of water pumps	Mise en place des pompes à eau									1	1	1	1	1	1	1	1	**8**
179	***Education***	***Education***																	**0**
180	Construction of schools by village with qualified teacher	Construction des écoles par village avec enseignant qualifié	1	1	1	1	1	1	1	1	1			1	1	1			**12**
181	Provision of school materials	Dotation de matériel scolaire										1	1				1	1	**4**
182	***Health***	***Santé***																	**0**
183	Availability of medicines and hospitals closer	Disponibilité des médicaments et des hôpitaux plus proche	1	1	1	1			1	1	1		1	1	1	1			**11**
184	Ease of processing partly free of charge	Facilité de traitement en partie gratuit					1		1								1		**3**
185	Competent doctor.	Docteur compétent.					1					1				1			**3**
186	***AGR***	***AGR***																	**0**
187	Sale of PPN	Vente de PPN									1		1	1		1		1	**5**
188	Laying hen	Poule pondeuse										1					1		**2**
189	Improvement of tobacco production	Amélioration de production de tabac													1				**1**

Overall

	FG1	FG2	FG3	FG4	FG5	FG6	FG7	FG8	FG9	FG10	FG11	FG12	FG13	FG14	FG15	FG16
FG1	1	0.5	0.59	0.58	0.45	0.51	0.46	0.48	0.36	0.34	0.28	0.32	0.37	0.37	0.27	0.28
FG2		1.0	0.64	0.56	0.49	0.51	0.54	0.56	0.38	0.32	0.26	0.38	0.45	0.34	0.32	0.31
FG3			1.00	0.64	0.52	0.60	0.54	0.58	0.27	0.27	0.25	0.34	0.41	0.33	0.33	0.28
FG4				1.00	0.45	0.51	0.53	0.55	0.30	0.35	0.31	0.35	0.38	0.36	0.29	0.25
FG5					1.00	0.67	0.70	0.64	0.33	0.35	0.21	0.33	0.40	0.40	0.39	0.29
FG6						1.00	0.65	0.73	0.32	0.32	0.26	0.37	0.43	0.38	0.34	0.29
FG7							1.00	0.77	0.40	0.38	0.34	0.45	0.43	0.43	0.42	0.30
FG8								1.00	0.43	0.43	0.40	0.46	0.49	0.48	0.39	0.34
FG9									1.00	0.67	0.71	0.69	0.62	0.59	0.49	0.49
FG10										1.00	0.69	0.58	0.44	0.43	0.55	0.43
FG11											1.00	0.66	0.53	0.52	0.51	0.51
FG12												1.00	0.52	0.56	0.47	0.58
FG13													1.00	0.75	0.69	0.62
FG14														1.00	0.61	0.58
FG15															1.00	0.66
FG16																1.00

Question 1: Did you feel a change in climate compared to the year or before 2000?

Dice-Sorensen coefficient matrix for group1 :

	FG1	FG2	FG3	FG4	FG5	FG6	FG7	FG8	FG9	FG10	FG11	FG12	FG13	FG14	FG15	FG16
FG1	1	1	1	1	1	1	1	1	1	1	1	1	1	1	1	1
FG2		1	1	1	1	1	1	1	1	1	1	1	1	1	1	1
FG3			1	1	1	1	1	1	1	1	1	1	1	1	1	1
FG4				1	1	1	1	1	1	1	1	1	1	1	1	1
FG5					1	1	1	1	1	1	1	1	1	1	1	1
FG6						1	1	1	1	1	1	1	1	1	1	1
FG7							1	1	1	1	1	1	1	1	1	1
FG8								1	1	1	1	1	1	1	1	1
FG9									1	1	1	1	1	1	1	1
FG10										1	1	1	1	1	1	1
FG11											1	1	1	1	1	1
FG12												1	1	1	1	1
FG13													1	1	1	1
FG14														1	1	1
FG15															1	1
FG16																1

Question 2: What have you noticed?

Dice-Sorensen coefficient matrix for group2 :

	FG1	FG2	FG3	FG4	FG5	FG6	FG7	FG8	FG9	FG10	FG11	FG12	FG13	FG14	FG15	FG16
FG1	1	0.24	0.4	0.60	0.67	0.60	0.36	0.50	0.44	0.44	0.22	0.22	0.44	0.40	0.25	0.25
FG2		1.00	0.4	0.27	0.24	0.27	0.38	0.35	0.43	0.43	0.29	0.29	0.29	0.27	0.31	0.31
FG3			1.0	0.62	0.53	0.62	0.71	0.80	0.50	0.50	0.50	0.50	0.50	0.62	0.36	0.36
FG4				1.00	0.60	0.75	0.67	0.80	0.57	0.57	0.29	0.57	0.57	0.75	0.33	0.33
FG5					1.00	0.80	0.55	0.67	0.44	0.44	0.22	0.22	0.44	0.40	0.25	0.25
FG6						1.00	0.67	0.80	0.57	0.57	0.29	0.29	0.57	0.50	0.33	0.33
FG7							1.00	0.91	0.75	0.75	0.50	0.75	0.50	0.67	0.57	0.57
FG8								1.00	0.67	0.67	0.44	0.67	0.67	0.80	0.50	0.50
FG9									1.00	1.00	0.67	0.67	0.67	0.57	0.80	0.80
FG10										1.00	0.67	0.67	0.67	0.57	0.80	0.80
FG11											1.00	0.67	0.67	0.57	0.80	0.80
FG12												1.00	0.67	0.86	0.80	0.80
FG13													1.00	0.86	0.80	0.80
FG14														1.00	0.67	0.67
FG15															1.00	1.00
FG16																1.00

Question 3: If there are any changes; In your opinion, what are the origins of the change?

Dice-Sorensen coefficient matrix for group3 :

	FG1	FG2	FG3	FG4	FG5	FG6	FG7	FG8	FG9	FG10	FG11	FG12	FG13	FG14	FG15	FG16
FG1	1	0.5	0.67	0.50	0.50	0.00	0.50	0.50	0.00	0.00	0	0.00	0.67	0.67	0.67	0.67
FG2		1.0	0.67	1.00	1.00	0.67	1.00	1.00	0.67	0.67	0	0.67	0.00	0.00	0.00	0.00
FG3			1.00	0.67	0.67	0.00	0.67	0.67	0.00	0.00	0	0.00	0.00	0.00	0.00	0.00
FG4				1.00	1.00	0.67	1.00	1.00	0.67	0.67	0	0.67	0.00	0.00	0.00	0.00
FG5					1.00	0.67	1.00	1.00	0.67	0.67	0	0.67	0.00	0.00	0.00	0.00
FG6						1.00	0.67	0.67	1.00	1.00	0	1.00	0.00	0.00	0.00	0.00
FG7							1.00	1.00	0.67	0.67	0	0.67	0.00	0.00	0.00	0.00
FG8								1.00	0.67	0.67	0	0.67	0.00	0.00	0.00	0.00
FG9									1.00	1.00	0	1.00	0.00	0.00	0.00	0.00
FG10										1.00	0	1.00	0.00	0.00	0.00	0.00
FG11											1	0.00	0.00	0.00	0.00	0.00
FG12												1.00	0.00	0.00	0.00	0.00
FG13													1.00	1.00	1.00	1.00
FG14														1.00	1.00	1.00
FG15															1.00	1.00
FG16																1.00

Question 4: What are the consequences of these changes?

Dice-Sorensen coefficient matrix for group4 :

	FG1	FG2	FG3	FG4	FG5	FG6	FG7	FG8	FG9	FG10	FG11	FG12	FG13	FG14	FG15	FG16
FG1	1	0	0.25	0	0.00	0.00	0.00	0.00	0.00	0.00	0.00	0.00	0.00	0.00	0.00	0
FG2		1	0.75	0	0.75	0.75	0.75	0.57	0.00	0.00	0.00	0.80	0.50	0.00	0.67	0
FG3			1.00	0	0.60	0.60	0.60	0.44	0.00	0.00	0.00	0.57	0.40	0.00	0.75	0
FG4				NaN	0.00	0.00	0.00	0.00	0.00	0.00	0.00	0.00	0.00	0.00	0.00	NaN
FG5					1.00	0.80	0.80	0.67	0.29	0.33	0.00	0.57	0.60	0.25	0.50	0
FG6						1.00	0.80	0.89	0.29	0.33	0.00	0.57	0.60	0.25	0.50	0
FG7							1.00	0.67	0.29	0.33	0.00	0.57	0.40	0.00	0.50	0
FG8								1.00	0.33	0.40	0.00	0.67	0.44	0.29	0.29	0
FG9									1.00	0.67	0.67	0.00	0.29	0.40	0.00	0
FG10										1.00	0.00	0.00	0.00	0.00	0.00	0
FG11											1.00	0.00	0.33	0.50	0.00	0
FG12												1.00	0.29	0.00	0.40	0
FG13													1.00	0.75	0.50	0
FG14														1.00	0.00	0
FG15															1.00	0
FG16																NaN

Question 5: What types of accommodations have you adopted?

Dice-Sorensen coefficient matrix for group5 :

	FG1	FG2	FG3	FG4	FG5	FG6	FG7	FG8	FG9	FG10	FG11	FG12	FG13	FG14	FG15	FG16
FG1	1	0.62	0.62	0.44	0.48	0.43	0.43	0.42	0.40	0.46	0.24	0.32	0.44	0.44	0.37	0.46
FG2		1.00	0.55	0.43	0.41	0.42	0.42	0.41	0.29	0.36	0.19	0.29	0.52	0.43	0.35	0.55
FG3			1.00	0.43	0.48	0.48	0.42	0.47	0.19	0.36	0.19	0.19	0.43	0.35	0.35	0.36
FG4				1.00	0.40	0.29	0.41	0.34	0.18	0.52	0.36	0.18	0.42	0.42	0.42	0.26
FG5					1.00	0.70	0.75	0.78	0.43	0.48	0.43	0.43	0.47	0.60	0.47	0.41
FG6						1.00	0.68	0.76	0.31	0.36	0.38	0.44	0.47	0.53	0.47	0.48
FG7							1.00	0.89	0.38	0.48	0.44	0.44	0.53	0.65	0.53	0.42
FG8								1.00	0.36	0.41	0.42	0.36	0.51	0.57	0.51	0.41
FG9									1.00	0.48	0.70	0.70	0.64	0.55	0.45	0.48
FG10										1.00	0.67	0.48	0.43	0.52	0.61	0.27
FG11											1.00	0.60	0.64	0.64	0.64	0.38
FG12												1.00	0.55	0.64	0.45	0.67
FG13													1.00	0.75	0.75	0.70
FG14														1.00	0.75	0.61
FG15															1.00	0.43
FG16																1.00

Question 6: What do you think are the gaps in resilience?

Dice-Sorensen coefficient matrix for group6 :

	FG1	FG2	FG3	FG4	FG5	FG6	FG7	FG8	FG9	FG10	FG11	FG12	FG13	FG14	FG15	FG16
FG1	1	0.59	0.67	0.57	0.37	0.59	0.55	0.58	0.36	0.29	0.29	0.29	0.28	0.37	0.21	0.14
FG2		1.00	0.72	0.78	0.45	0.62	0.58	0.77	0.35	0.26	0.26	0.26	0.42	0.36	0.26	0.17
FG3			1.00	0.92	0.40	0.69	0.52	0.62	0.15	0.15	0.15	0.23	0.30	0.24	0.23	0.22
FG4				1.00	0.43	0.67	0.56	0.67	0.17	0.17	0.17	0.25	0.32	0.26	0.25	0.24
FG5					1.00	0.55	0.67	0.46	0.09	0.00	0.00	0.09	0.25	0.27	0.35	0.25
FG6						1.00	0.58	0.67	0.20	0.13	0.13	0.20	0.32	0.28	0.20	0.13
FG7							1.00	0.50	0.24	0.16	0.16	0.24	0.23	0.25	0.24	0.15
FG8								1.00	0.37	0.44	0.37	0.37	0.43	0.38	0.30	0.29
FG9									1.00	0.83	0.83	0.75	0.64	0.61	0.58	0.48
FG10										1.00	0.92	0.83	0.48	0.43	0.42	0.48
FG11											1.00	0.83	0.48	0.43	0.42	0.48
FG12												1.00	0.48	0.52	0.50	0.56
FG13													1.00	0.83	0.72	0.69
FG14														1.00	0.78	0.67
FG15															1.00	0.88
FG16																1.00

Question 7. Proposal of solutions

Dice-Sorensen coefficient matrix for group7 :

	FG1	FG2	FG3	FG4	FG5	FG6	FG7	FG8	FG9	FG10	FG11	FG12	FG13	FG14	FG15	FG16
FG1	1	0.57	0.67	0.88	0.50	0.80	0.57	0.57	0.38	0.25	0.40	0.50	0.40	0.25	0.13	0.14
FG2		1.00	0.91	0.67	0.67	0.55	0.60	0.60	0.50	0.17	0.36	0.50	0.55	0.33	0.18	0.20
FG3			1.00	0.62	0.62	0.67	0.55	0.55	0.46	0.15	0.33	0.46	0.50	0.31	0.17	0.18
FG4				1.00	0.43	0.62	0.67	0.67	0.43	0.14	0.46	0.57	0.46	0.29	0.15	0.17
FG5					1.00	0.62	0.50	0.33	0.29	0.43	0.15	0.29	0.31	0.29	0.31	0.17
FG6						1.00	0.55	0.55	0.31	0.31	0.33	0.31	0.33	0.15	0.17	0.18
FG7							1.00	0.80	0.50	0.17	0.55	0.50	0.55	0.33	0.36	0.20
FG8								1.00	0.50	0.17	0.55	0.50	0.55	0.33	0.18	0.20
FG9									1.00	0.43	0.62	0.71	0.77	0.71	0.46	0.50
FG10										1.00	0.46	0.29	0.46	0.29	0.77	0.50
FG11											1.00	0.62	0.50	0.46	0.50	0.73
FG12												1.00	0.62	0.57	0.31	0.50
FG13													1.00	0.46	0.50	0.36
FG14														1.00	0.15	0.33
FG15															1.00	0.55
FG16																1.00
